# Prevalence of extended spectrum Beta-Lactamase producing *Klebsiella* species from patients' specimens in a tertiary teaching hospital in Ile-Ife, Southwest Nigeria

**DOI:** 10.4314/ahs.v22i2.17

**Published:** 2022-06

**Authors:** Omolola Esther Ajimuda, Ikeoluwa Sanmi-Kayode, Oluwagbeminiyi Oyinkansola Adeniyi, Olubunmi Olayemi Alaka, Anthony Onipede

**Affiliations:** 1 Department of Medical Microbiology and Parasitology, Obafemi Awolowo University, Ile-Ife, Nigeria; 2 Department of Medical Microbiology and Parasitology, Obafemi Awolowo University Teaching Hospitals Complex, Ile-Ife, Nigeria

**Keywords:** Antibiotic Resistance, *Klebsiella*, ESBL

## Abstract

**Objective:**

This study determined the prevalence of ESBL genes amongst *Klebsiella* species isolated from patients' specimens attending a Tertiary Teaching Hospital, in Ile-Ife, Southwest Nigeria.

**Methods:**

A cross sectional study of presumptive isolates of *Klebsiella* (n=180) were collected, after ethical approval in the microbiology laboratory. Isolates were identified to species level by conventional biochemical tests and MicrobactTM 24E Identification Kits. Antibiotic susceptibility testing was performed by the Kirby Bauer's disk diffusion method. ESBL production was detected by the double disc synergy and ESBL genes by the multiplex PCR protocol.

**Results:**

The *Klebsiella* species identified were *Klebsiella pneumoniae* 95.6%, *Klebsiella oxytoca* 3.3%, *Klebsiella ornithinolytica* 0.6% and *Klebsiella terrigena* 0.6%. The prevalence of ESBL genes among the *Klebsiella* isolates was 47.2%, and the most common ESBL gene was blaSHV (38.9%), also 75% of the study isolates had MAR index greater than 0.2.

**Conclusions:**

The study establishes the prevalence of Extended Spectrum Beta-Lactamase producing Klebsiella sp in the hospital and identified the most prevalent ESBL genes circulating as blaSHV followed by blaTEM and blaCTX-M in this environment. This underscores the need for regular and continuous surveillance of antimicrobial resistance trend as a strong component of the antibiotics stewardship and infection prevention and control programs in the hospital.

## Introduction

*Klebsiella* species are mostly opportunistic pathogens found commonly in people with other comorbid infections, they are also able to act as primary pathogens in otherwise healthy individual causing urinary tract infections and pneumonia for example. They have played major role in aggravating the issue of antibiotic resistance by being multidrug resistant in most cases and being able to transfer the resistant genes to other organisms and they have been recognized as a persistent public health problem according to WHO. 1

The increase in the incidence of extended-spectrum β-lactamase- (ESBL-) producing *Klebsiella* species have become a serious challenge in healthcare delivery globally. Genes conferring these resistances which are mediated by enzymes known as extended spectrum beta-lactamases (ESBLs) are wide spread among several Enterobacteriaceae species. The resistance in the ESBL producing *Klebsiella* spp is associated with acquired extended-spectrum beta lactamase (ESBL) genes located on mobile genetic elements such as plasmids or transposons, that codes for Beta lactamases such as CTX-M, TEM and SHV, which are the most clinically significant ones, others include OXA and PE^2^. Owing to the increasing epidemiological and therapeutic challenges associated with infections due to ESBL producers among some bacteria isolates, there is increased reports of ESBL dissemination from various centers in south western, Nigeria among Enterobacteriaceae organisms particularly *Klebsiella pneumoniae* isolates.^3^ This is a concern both to clinicians and other health practitioners due to the obvious consequences of treatment failures, prolonged patients' stay in the hospital and nosocomial spread of resistant organisms. Geser et al. identified selective pressure resulting from the indiscriminate use of the extended spectrum cephalosporins both in therapeutic and sub-therapeutic doses amongst other factors as responsible for the observed resistance isolates^4^.

*Klebsiella* species is resistant to many antibiotics using various means including point mutations in quinolones and production of genes such as ESBLs and KPC that hydrolyze antibiotics^5^. Various studies have shown the significance and prevalence of ESBL genes, Vasaikar *et al.* reported a 60.4% prevalence of ESBL producers in a study on the resistance profile of *Klebsiella* isolates in Eastern Cape Province, South Africa.^6^

The worldwide increase in infections caused by extended-spectrum beta-lactamase- (ESBL) and AmpC-producing Enterobacteriaceae (ESBL-E) is a concern. Surveillance is extensive in Europe, North America, and Asia. Yet, there is no summarizing surveillance in Africa.^7^ Resistance to antibiotics most especially third generation cephalosporins has assumed a worrisome dimension globally. Genes conferring this resistance which are mediated by enzymes known, as extended spectrum beta-lactamases (ESBLs) are now wide spread among several Enterobacteriaceae species.^8^ This study was therefore designed to determine the prevalence of ESBL genes amongst *Klebsiella* species isolated from patients' specimens attending Obafemi Awolowo University Teaching Hospitals Complex, Ile-Ife, Nigeria as a baseline surveillance effort.

## Methodology

### Study site

The study was carried out at the Department of Medical Microbiology and Parasitology of Obafemi Awolowo University and Teaching Hospital Complex, Ile-Ife, Osun State, South Western Nigeria.

### Study design

This was a hospital based, cross sectional study of specimens isolates of *Klebsiella* spp from patients undergoing care at the Teaching hospital in Ile-Ife, Nigeria.

### Ethical Approval

Approval was obtained from the Ethics and Research committee of Obafemi Awolowo University Teaching Hospital Complex, Ile-Ife, Nigeria.

### Sample size and isolate collection

The isolates used for this study were collected from processed clinical specimens from the teaching hospital medical microbiology and parasitology laboratory. A total of 180 non-repetitive *Klebsiella* isolates were collected from routine clinical specimens from September 2018- February 2019.

### Processing of samples

Bacterial identification: Isolates were re-inoculated on Mac Conkey agar (Hi media labs) and incubated aerobically for 24 hours at 37°C. The isolates were first identified using Gram's reaction, Oxidase reaction using oxidase strips, citrate utilization, sulphide, indole and motility reactions using conventional methods. All isolates were then confirmed using the Microbact 24E Identification Kits.

### Antimicrobial susceptibility testing

Antimicrobial susceptibility testing of each isolate was performed by using Kirby-Bauer disc diffusion technique using Mueller Hinton agar (Oxoid Ltd., Basingstoke, Hampshire, England) according to the guidelines of Clinical and Laboratory Standards Institute^9^. The Antimicrobial discs (ug) that were used are Amoxycillin (10ug), Amoxycillin/ Clavulinic Acid (20/10ug), Cefoxtin (30ug, Cefotaxime(30ug), Cefepime(30ug), Gentamicin(10ug), Ciprofloxacin (5ug), Chloramphenicol (30ug), levofloxacin(5ug), Nalixidic acid (30ug), and meropenem(10ug). E. coli ATCC 29522 served as the quality control strain.

### Extended spectrum β lactamase screening

Tests for ESBL production: Test for ESBL production was done using double disc synergy method according to EUCAST.^10^

### Double disc synergy method

Antibiotic discs of amoxicillin/ clavulinic acid (20/10 ug) was put in between ceftazidime (30ug) and cefotaxime (30 ug) at a distance of 20 mm apart and incubated. Isolates that showed a clear zone of extension of either of ceftazidime or cefotaxime inhibition zone towards the amoxicillin/ clavulinic acid (20/10 ug) disc was considered ESBL producers. E. coli ATCC 25922 was used as negative controls.

### Genotypic detection of ESBLs

DNA was extracted from *Klebsiella* isolates suspected to be ESBL producers from phenotypic detection using the boiling method and screened by polymerase chain reaction using primers specific for the detection of blaCTX-M, blaTEM and blaSHV genes^11^.

### Beta-lactamase genes amplification^11^

All *Klebsiella* isolates that were phenotypically positive for ESBL production were screened by PCR using primers specific for the detection of blaSHV, blaTEM, and blaCTX-M genes. PCR amplifications of blaSHV, blaTEM, and blaCTX-M genes were performed in 25 µl reaction mixes containing 25 units/ml of Taq DNA polymerase, 200 µM each of dATP, dGTP, TTP and dCTP, 0.2 µM of each primer, 1.5 mM MgCl2 and 5 µl of total DNA template. Isolate's DNA was amplified under the following conditions: initial denaturation at 94°C for 3 minutes, followed by 35 cycles of denaturation at 94°C for 45 seconds, annealing at 60°C for TEM, SHV and CTX-M and extension at 72°C for 1 minute, and a final extension at 72°C for 3 minutes. Escherichia coli ATCC 25922 was used for negative control, and PCR products (10 µl of each)were prepared and detected using gel electrophoresis as previously described^11^.

### Data analysis

Data analysis was done using statistical package for social sciences (SPSS) version 26.0^12^.

## Results

A total of 180 *Klebsiella* species isolates were obtained, and of the 180 isolates *Klebsiella pneumoniae* was (172) 95.6%, *Klebsiella oxytoca* (6) 3.3%, *Klebsiella ornithinolytica* (1) 0.5% and Klebsiella terrigena (1) 0.5%. Their specimen source is reflected in Table 2. The antibiotic resistance profile of the isolates is presented in Table 3, the *Klebsiella* isolates were mostly resistant to Ampicillin (159) 88.3% but were least resistant to meropenem (4) 2.2 %). The multiple antibiotic resistance (MAR) index and patterns for the isolates are presented in Table 4. Table 5 shows that 47.2% of the isolates carried ESBL gene while 52.8% did not carry any gene. blaSHV gene was the most commonly carried ESBL gene in 70 (38.9%) isolates followed by blaTEM in 54 (30%) and blaCTX-M in 2 (23.3%) isolates (Fig 1.0). Hospitalization (p<0.008), use of Medical device (p<0.009), resistance to more than three classes of antibiotics (p<0.001) were significantly associated with ESBL *Klebsiella* infection at p<0.05. The result of analysis is shown in Table 6. Table 75 shows the antibiotics resistance patterns of the isolates, ampicillin was the most common antibiotics in the resistance pattern, and MAR index 0.36 has the highest varying pattern, and meropenem was the least common antibiotics with resistance. Table 8 shows the ESBL producing *Klebsiella* sp and their varying MAR index.

**Table 2:** *Klebsiella* isolates and their sample sources

	Urine n (%)	Swab n (%)	Aspirate n (%)	Sputum n (%)	Stool n (%)	Blood n (%)	Other n (%)	Total n (%)
*K.pneumoniae*	86(47.8)	n (%) 36(20)	n (%) 4(2.2)	n (%) 13(7.2)	n(%) -	n (%) 27(15.0)	n(%) 6(3.3)	n(%) 172(95.6)
*K.oxytoca*	4(2.2)	1(0.6)	-	-	1(0.6)	-	-	6(3.3)
*K.terrigena*	-	-	-	1 (0.6)	-	-	-	1 (0.6)
*K.ornithinolytica*	1(0.6)	-	-	-	-	-	-	1(0.6)
Total	91(50.6)	37(20.6)	4(2.2)	14(7.8)	1(0.6)	27(15.0)	6(3.3)	180(100)

N= number of isolates

**Table 3:** Antimicrobial resistance rates in the Klebsiella isolates

Antibiotics	*Klebsiella* spp				Total N=180
					
	*K.pneumoniae* (n=172 (%))	*K.oxytoca* (n=6 (%))	*K.ornithinolytica* (n=1(%))	*K.terrigena* (n=1(%))	
Ampicillin	151(87.8)	6(100.0)	1(100.0)	1(100.0)	159(88.3)
Amoxicillin/Clavulanic Acid	60(34.8)	0(0.00)	0(0.0)	0(0.0)	60(33.3)
Cefoxitin	90(52.3)	3(50.0)	1(100.0)	1(100.0)	95(52.8)
Cefotaxime	105(61.1)	2(33.3)	1(100.0)	1(100.0)	109(60.6)
Cefepime	88 (51.2)	0(0.0)	0(0.0)	1(100.0)	89(49.4)
Chloramphenicol	65(37.8)	1(16.7)	0(0.0)	1(100.0)	67(37.2)
Nalixidic Acid	87(50.6)	2(33.3)	0(100.0)	1(100.0)	90(50.0)
Levofloxacin	100(58.1)	2(33.3)	0(0.0)	1(100.0)	103(57.2)
Ciprofloxacin	108(62.8)	2(33.3)	0(100.0)	1(100.0)	111(61.7)
Gentamicin	90(52.3)	1(16.7)	0(0.0)	0(0.0)	91(50.6)
Meropenem	4(2.3)	0(0.0)	0(0.0)	0(0.0)	4(2.2)

N= number of isolates

**Table 4:** MAR indices of *Klebsiella* species (n=180)

MAR Index	Number (%)
0	2 (1.1)
0.09	21(11.7)
0.18	21(11.7)
0.27	15(8.3)
0.36	20(11.1)
0.45	9(5.0)
0.55	14(7.8)
0.64	17(9.4)
0.73	23(12.8)
0.82	22(12.2)
0.91	15(8.3)
1	1(0.6)

**Table 5:** Prevalence of ESBL genes in *Klebsiella* Isolates

ESBL genes	*K. pneumoniae* (%)	*K.oxytoca* (%)	*K.terrigena* (%)	*K.ornithinolytica* (%)	Total (%)
*bla*CTX-M	3 (1.7)	-	-	1 (0.6)	4(2.2)
*bla*SHV	16 (8.9)	1(0.6)	-	-	17(9.4)
*bla*TEM	8 (4.4)	-	-	-	8(4.4)
*bla*CTX-M/*bla*SHV	6 (3.3)	1(0.6)	1(0.6)	-	8(4.4)
*bla*SHV/*bla*TEM	18 (10.0)	-	-	-	18(10.0)
*bla*CTX-M/*bla*TEM	3 (1.7)	-	-	-	3(1.7)
*bla*CTX-M/*bla*TEM/*bla*SHV	27 (15.0)	-	-	-	27(15.0)
No ESBL gene	91 (50.6)	4(2.2)	-	-	95(52.8)
Total	172(95.6)	6(3.3)	1(0.6)	1(0.6)	180(100.0)

**Figure 1:**
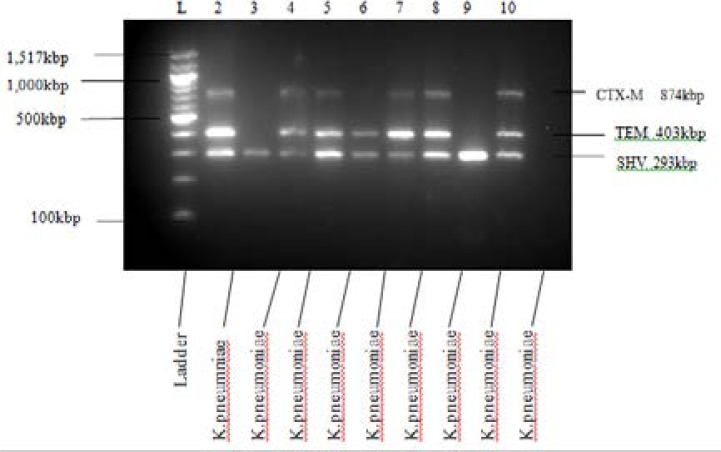
0 ESBL genes detection with Gel Electrophoresis

**Table 6:** Socio-Demographic data, Hospitalization records, Antibiotics use and Risk factors Associated with infection caused by ESBL producing *Klebsiella* Spp.

Risk Factors	ESBL +VE (85)(%)	ESBL-VE (95)(%)	χ^2^(p-value)
Age group			
0–1	7(3.9%)	6(3.3%)	0.043(0.835)
2–18	6(3.3%)	14(7.8%)	1.957 (0.162)
19–35	26(14.4%)	25(13.9%)	0.220(0.639)
35–60	27(15.0%)	23(12.8)	0.927(0.336)
>60	19(10.6%)	27(15.0%)	0.579(0.447)
Mean age 15.2			
IQR 35			
Gender			
Male (80)	37 (20.6%)	43 (23.9%)	0.007(0.933)
Female (100)	48(26.7%)	52(28.9%)	
Educational studies			0.503(0.478)
No& Pri (28)	11(6.1%)	17(9.4%)	
Sec and Ter (152)	74(41.1)	78(43.3%)	
Hospitalization			7.041(0.008)*
Yes (128)	69(38.3)	59(32.8)	
No (52)	16(8.9)	36(20.0)	
Medical device			6.794(0.009)*
Yes (126)	68(37.8)	58(32.2)	
No(54)	17(9.4)	37(20.6)	
Past antibiotic use			0.014(0.906)
Yes (91)	50(27.8)	54(30.0)	
N0 (89)	35(19.4)	41(22.8)	
Multiple resistance			
≥R3	76(42.2)	59(32.8)	16.414(<0.001)*
<R3	9(5.0)	36(20.0)	

**Table 7:** Resistance patterns of Klebsiella isolates according to their MAR indices

MAR index	Resistance patterns
0	
0.09	LEV; AMP; FOX
0.18	CTX, AMP; C, AMP; LEV, CIP; CIP, AML; AMC, FOX; FEP, AML; CTX, FOX
0.27	CTX, AML, FOX; FEP, CTX, AML;CTX, C, AML; CTX, NA, AML; C, AML,FOX; CIP, AML, FOX; AMC, AML,FOX.
0.36	NA,CIP,LEV,AML; C,NA,CIP,LEV; CIP,LEV,GEN,AML; FEP, CTX, AML, LEV; FEP,CTX,NA,LEV; FEP,C,GEN,AML; FEP,CTX,AML,FOX; FEP,CTX,GEN,FOX; CTX,C,AML,FOX;CTX,AMC,AML,FOX;C,CIP,AML,FOX; CIP,AMC,GEN,AML; CIP,GEN,AML,FOX.
0.45	NA, LEV,CIP,GEN,AML; NA,LEV,CIP,AMC,AML; LEV,CIP,GEN,AML,FOX; NA,LEV,CIP,AML; FEP,CTX,NA,CIP,AMC; FEP,CTX,CIP,GEN,AML;FEP,CTX,GEN,AML,FOX;
0.55	FEP,CTX,C,LEV,CIP,AML; FEP,CTX,LEV,CIP,GEN,AML;FEP,NA,LEV,CIP,GEN,FOX; CTX,NA,LEV,CIP,AML,FOX; C,NA, LEV,CIP,GEN,AML; C,NA,LEV,CIP,AML,FOX;C,NA,LEV,CIP,GEN,AML; NA,LEV,CIP,AMC, GEN,AML; FEP,CTX,C,AMC,GEN,AML;FEP,CTX,CIP,GEN,AML,FOX;FEP,CTX,AM C,GEN,AML,FOX.
0.64	FEP,CTX,NA,LEV,CIP,GEN,AML; FEP,CTX,C,LEV,CIP,GEN,AML; FEP,CTX,NA,LEV,CIP,AML,FOX; CTX,NA,LEV,CIP,AMC,AML,FOX; CTX,C,NA,LEV,CIP,AML,FOX; C,NA,LEV,CIP,GEN,AML,FOX; C,NA,LEV,CIP,AMC,AML,FOX; FEP,CTX,CIP,AMC,GEN,AML,FOX.
0.73	FEP,CTX,C,NA,AMC,AML,MER,FOX; C,NA,LEV,CIP,AMC,GEN,AML, FOX; FEP,C,NA,LEV,CIP,GEN,AML,FOX; FEP,CTX,C,NA,LEV,CIP,AML,FOX; FEP,CTX,C,NA,LEV,CIP,GEN,AML; FEP,CTX,C,LEV,CIP,AMC,GEN,AML; FEP,CTX,NA,LEV,CIP,AMC,GEN,AML; FEP,CTX,NA,LEV,CIP,AMC,AML,FOX; FEP,CTX,LEV,CIP,AMC,GEN,AML,FOX; FEP,CTX,NA,LEV,CIP,GEN,AML,FOX
0.82	FEP,CTX,C,NA,LEV,CIP,AMC,GEN,AML,FOX; FEP,CTX,C,NA,LEV,CIP,AMC,GEN,AML; FEP,CTX,C,NA,LEV,CIP,AMC,AML,FOX; FEP,CTX,C,NA,LEV,CIP,GEN,AML,FOX; FEP,CTX,C,LEV,CIP,AMC,GEN,AML,FOX; FEP,CTX,NA,LEV,CIP,AMC,GEN,AML,FOX; FEP,CTX,NA,LEV,CIP,AMC,AML,MER,FOX; CTX,C,NA,LEV,CIP,AMC,GEN,AML,FOX.
0.91	FEP,CTX,C,NA,LEV,CIP,AMC,GEN,AML,FOX; FEP,CTX,NA,LEV,CIP,AMC,GEN,AML,MER,FOX
1	FEP,CTX,C,NA,LEV,CIP,AMC,GEN,AML,MER,FOX

**Table 8:** ESBL production and MAR index

*Klebsiella* specie	ESBL producers and Resistance to antibiotics class No (%)								Total No %)
	
	0.36	0.45	0.55	0.64	0.73	0.82	0.91	1	
*K.pneumoniae*	4(2.2)	4(2.2)	6(3.3)	13(7.2)	16(8.9)	17(9.4)	12(6.7)	1(0.6)	73(40.6)
*K.oxytoca*	0	0	1(0.6)	0	0	0	0	0	1(0.6)
*K.ornithinilytica*	1(0.6)	0	0	0	0	0	0	0	1(0.6)
*K.terrigena*	0	0	0	0	1(0.6)	0	0	0	0(0.6)
Total	5(2.8)	4(2.2)	7(3.9)	13(7.2)	17(9.4)	17(9.4)	12(6.7)	1(0.6)	76(42.2)

## Discussion

In most studies conducted on the genus *Klebsiella, Klebsiella pneumoniae* has consistently being recognized as the predominant species6,, which is also evident in this study where 95.6% of the clinical isolates were identified as *Klebsiella pneumoniae*, followed by *Klebsiella oxytoca* (3.3%). Also to a lesser extent species which are not common clinical pathogens were isolated in this study including *Klebsiella terrigena* isolated from sputum in a patient visiting the renal ward who was diagnosed of pulmonary tuberculosis and *Klebsiella ornithinolytica* isolated from urine, both of which constituted 1.1% of the entire population of isolates studied. These uncommon isolates will be understood in the context of the underlying chronic clinical conditions of the patient with negative impact on their immune status or as a consequence thereof.

The reported distribution of *Klebsiella pneumoniae* in comparison to other Klebsiella spp in this study is closely in-line with that of Dallal et al.,^13^ who reported Klebsiella pneumoniae of (94%), and Klebsiella oxytoca (4%) in their study, but differs from that of Thonda and Oluduro^14^, who reported *Klebsiella pneumoniae* of (46%), *Klebsiella oxytoca* (19%), and *K. ornithinolytica* (6%). This observation may be due to differences in study population and sample source. *Klebsiella's* interaction with human ranges from asymptomatic carriage to opportunistic infections, also including community-acquired infections like UTI, bacteremias, upper and lower respiratory tract, surgical wound sites, pneumonia, diarrhea, and wound infection^15^ most of which are evident in this current study as shown in Table 2. Isolation of *Klebsiela pneumoniae* in samples such as blood and urine which are normally sterile under healthy conditions is an indication of their aetio-pathogenic roles at these sites considering the fact that most of the patients with these isolates were retrieved from those who were either on admission or discharged for follow up or were placed on medical devices while on admission. There were also cases of UTI due to bladder outlet obstruction because of Benign Prostatic Hyperplasia (BPH). It has been established that a well-functioning Infection Prevention and Control Program can go a long way to disrupt the chains of transmission, however, inadequate efforts in this direction can aid the rapid spread of resistant *Klebsiella* isolates. This may also explain the underlying factors identified for the number of *Klebsiella isolates* from blood samples (15%) of some patients who had sepsis due to primary diagnosis such as end stage renal disease, liver cirrhosis or heart tumours all in need of hospital care and optimum nosocomial prevention practices^4^,^5^.

Resistance to antibiotics has been widely reported in hospitalized patients with *Klebsiella* infections^1^,^2^. *Klebsiella pneumoniae* is known to have intrinsic resistance to a number of antibiotics which may partially explain the high level of resistance observed in this study coupled with the ESBL genes at play; for example resistance to ampicillin was (88.3%) in this study which correlates fairly with that of Mansouri et al.^16^ who reported (84%) for their study, though it was resistance to amoxicillin, a broad spectrum antibiotics that was tested. Furthermore, *Klebsiella* isolates were found to be resistant to third generation cephalosporins as well, cefotaxime (60.6%) resistance was high compared to other classes of cephalosporin tested, and was used as a surrogate-marker for the presence of ESBL producers in the population of *Klebsiella* isolates studied. For quinolone and fluoroquinolone, the quinolone (nalidixic acid) tested proved to be less resistant compared to the fluoroquinolones (levofloxacin and ciprofloxacin), though an unexpected finding, however, it could be explained because nalidixic acid is not commonly used again in the study area; therefore, there is reduced pressure on its use or a different mechanism is at play. It has been well established that three mechanisms of resistance to quinolones are currently recognized, which are mutations that alter the drug targets, mutations that reduce drug accumulation, and plasmids that protect cells from the lethal effects of quinolones^17^. In this instance, one can speculate that there is no altered drug target for Nalidixic acid in the present study, however, these calls for more cross-resistance studies to identify the mechanism involved which may influence treatment alternatives based on resistance profiles^18^. *Klebsiella* isolates in this study retained high susceptibility to meropenem being a relatively new drug and not available over the counter without a doctor's prescription. Meropenem, a carbapenem-type broad-spectrum antibiotic used in treating a variety of bacterial infections proved to be the most sensitive antibiotics in this study, which according to Tamma, et al. is now recognized as the gold standard for treatment of infections caused by ESBL producing organisms including *Klebsiella pneumoniae*.^19^

Comparing our results to another study^14^ carried out in the same area, the resistance rates obtained in this study were lower for ampicillin, amoxicillin/clavulanic acid and meropenem, but were higher for cefoxitin, cefotaxime, chloramphenicol, ciprofloxacin and gentamicin. The increase in these resistance rates is an indication of selective pressure on these drugs and the consequent spread of antibiotic resistant pathogens in both the hospital and community in the absence of an effective antibiotics resistance surveillance and control programme in place.

ESBL producing *Klebsiella* has been associated with mortality and treatment failure, in this study we recorded a prevalence of (47.2%) for ESBL production amongst *Klebsiella* species which we consider high, the most common ESBL gene identified was blaSHV (38.9%), followed by blaTEM (29.4%) and blaCTX-M (23.3%) which was the least observed. Only 27(15%) of the entire population of *Klebsiella* isolates carried all three genes at once. This finding is consistent with the report from north-eastern Nigeria, where blaSHV (36.4%) was also the highest in their series followed by TEM (31.4%) and CTX-M (27.3%)^20^ but differs from that reported previously in south-western Nigeria14 from the same study area as the current study, where blaTEM was the most prevalent ESBL gene identified. This observation could imply a change in trend of genes responsible for resistance due to ESBL production or differences in types of isolates studied. Most ESBL-producing enterobacterial isolates are at the same time multidrug resistant^21^, 75% of the *Klebsiella* species. Previous Admission to health care facility and antibiotic treatment amongst other factors are recognized risk factors for acquiring ESBL producing *Klebsiella* spp^14^,^20^. Of all the risk factors tested statistically only three were significant which are: Hospitalization (p=0.008), use of Medical device (p=0.009) and Multiple Antibiotics Resistance (MAR) index (p<0.001). This significant association could be due to the nosocomial spread of this strain of bcteria within the health facility following excessive pressure of antibiotics usage in hospitalized patients. The role of hospitalization, medical devices and previous antibiotics as risk factors for acquisition of ESBL pathogens have also been reported in an earlier study by Demirdag and Hosoglu^23^, as also significant factors in the spread of ESBL pathogens.

Although ESBL producing *Klebsiella* infection has been associated with increase in length of hospital stay and additional treatment costs resulting from expensive alternative treatments. Duration of hospitalization, a tool which would have been useful in measuring the increase in days of hospitalization as well as extra treatment cost was not investigated in this study, although it has been well established that the longer the duration of hospitalization the higher the risk of colonization with circulating hospital resistant strain of pathogens^24^.

## Conclusion

This study revealed that ESBL production and multidrug resistance are relatively high in *Klebsiella* isolates circulating in the hospital environment and identified the most prevalent ESBL genes circulating as blaSHV followed by blaTEM and blaCTX-M as the least circulating in this environment. All ESBL-producing isolates demonstrated multidrug resistance to antimicrobial agents belonging to at least three different classes of antimicrobials. This poses a great threat to the effective treatment of patients with ESBL producing *Klebsiella* infection and the associated increased risk of mortality and health care cost. This calls for a review of the current treatment policy/guidelines of this group of organisms and underscores the need for regular and continuous surveillance of antimicrobial resistance trend as a strong component of the antibiotics stewardship and infection prevention and control programs in the hospital.

## Figures and Tables

**Table 1:** ESBL primers nucleotide sequence and size

ESBL gene		Nucleotide sequence	Size (kbp)
SHV	SHV-F	CGCCTGTGTATTATCTCCCT	293
	SHV-R	CGAGTAGTCCACCAGATCCT	
TEM	TEM-F	TTTCGTGTCGCCCTTATTCC	403
	TEM-R	ATCGTTGTCAGAAGTAAGTTGG	
CTX-M	CTX-M-F	CGCTGTTGTTAGGAAGTGTG	874
	CTX-MR	GGCTGGGTGAAGTAAGTGAC	
